# Exposure to the non-phthalate plasticizer di-heptyl succinate is less disruptive to C57bl/6N mouse recovery from a myocardial infarction than DEHP, TOTM or related di-octyl succinate

**DOI:** 10.1371/journal.pone.0288491

**Published:** 2023-07-13

**Authors:** Adam Schwendt, Joey-Bahige Chammas, Milan Maric, Jim A. Nicell, Richard Leask, Lorraine E. Chalifour

**Affiliations:** 1 Lady Davis Institute for Medical Research, Montréal, Québec, Canada; 2 Department of Chemical Engineering, Faculty of Engineering, McGill University, Montréal, Québec, Canada; 3 Department of Civil Engineering, Faculty of Engineering, McGill University, Montréal, Québec, Canada; 4 Division of Experimental Medicine, Faculty of Medicine and Health Sciences, McGill University, Montréal, Québec, Canada; Kimura Hospital, JAPAN

## Abstract

Phthalate plasticizers are incorporated into plastics to make them soft and malleable, but are known to leach out of the final product into their surroundings with potential detrimental effects to human and ecological health. The replacement of widely-used phthalate plasticizers, such as di-ethylhexyl phthalate (DEHP), that are of known toxicity, by the commercially-available alternative Tris(2-ethylhexyl) tri-mellitate (TOTM) is increasing. Additionally, several newly designed “green” plasticizers, including di-heptyl succinate (DHPS) and di-octyl succinate (DOS) have been identified as potential replacements. However, the impact of plasticizer exposure from medical devices on patient recovery is unknown and, moreover, the safety of TOTM, DHPS, and DOS is not well established in the context of patient recovery. To study the direct effect of clinically based chemical exposures, we exposed C57bl/6 N male and female mice to DEHP, TOTM, DOS, and DHPS during recovery from cardiac surgery and assessed survival, cardiac structure and function, immune cell infiltration into the cardiac wound and activation of the NLRP3 inflammasome. Male, but not female, mice treated *in vivo* with DEHP and TOTM had greater cardiac dilation, reduced cardiac function, increased infiltration of neutrophils, monocytes, and macrophages and increased expression of inflammasome receptors and effectors, thereby suggesting impaired recovery in exposed mice. In contrast, no impact was detected in female mice and male mice exposed to DOS and DHPS. To examine the direct effects in cells involved in wound healing, we treated human THP-1 macrophages with the plasticizers *in vitro* and found DEHP induced greater NLRP3 expression and activation. These results suggest that replacing current plasticizers with non-phthalate-based plasticizers may improve patient recovery, especially in the male population. In our assessment, DHPS is a promising possibility for a non-toxic biocompatible plasticizer.

## Introduction

Plastics are ubiquitous in the environment because they are important in the manufacture and storage of food, personal care products and a myriad of other products used in everyday life [1]. People are exposed to plastics and the chemicals which leach from plastics via oral and dermatological routes, and in the air from household dust and the soil as they degrade. Plasticizers, such as di-ethylhexyl phthalate (DEHP) which serves to make hard poly(vinyl) carbonate (PVC) soft and pliable, can form a high percentage of the final product, are not chemically bound to PVC and escape from the plastic to contaminate the contents of the plastic containers [2]. Many studies have explored the impact of exposure to DEHP and other plasticizers on human and animal health [3]. It is now recognized that DEHP is a Substance of Very High Concern because of its recognized reproductive toxicity and endocrine disruption properties. Early life exposure to DEHP disrupts the signaling of male and female hormones and negatively influences the development of reproductive organs in progeny [1]. Some studies found DEHP-induced changes in the prostate cells associated with an increased susceptibility to prostate cancer development [4]. In humans, epidemiological analyses have not found a clear association between increased DEHP exposure and increased cancer [5]. However, increased DEHP metabolites were correlated with increased prostate cancer in American men [6] and in obese Taiwanese men [7]. Associations between increased DEHP metabolites and breast cancer may be ethnicity-dependent. Increased DEHP metabolite detected was lined with an increased risk of breast cancer in subpopulations in a Multiethnic Cohort study [8], in Indian women [9] but not in the Women’s Health Initiative study of American women [10]. As a result of these and other findings, DEHP is banned in childcare items and the amount of DEHP permissible in plastic items is limited to 0.1% by weight.

Plastics and plastic components make up the majority of medical devices [11–14]. As in food and other containers, phthalates are added to confer softness and flexibility to the otherwise hard and rigid PVC materials used in medical devices. Leaching of phthalates from the plastic device into the patient is measureable. In the adult ICU, urine phthalate metabolites increased 100- to 1000-fold [15] and serum metabolites increased from ~103 ng/ml to ~2100 ng/ml post-surgery over pre-surgery levels [16]. Previously in our lab, we found the metabolites of 10 different phthalates in patient urine were massively increased relative to pre-surgery levels revealing device-related exposure to DEHP (~1,600-fold), butyl benzyl phthalate (BBzP, ~5,100-fold), di-*n*-butyl phthalate (DnBP, ~55-fold) and di-isodecyl phthalate (DIDP,~95-fold) shortly after cardiac surgery [17]. Bastiaensen *et al*. detected multiple phthalates, alternative plasticizers and phosphate flame retardants in samples collected from an adult intensive care unit population and linked the increases to medical devices [18]. Similarly, use of increased numbers of medical devices was associated with greater phthalate exposure in samples from patients in the neonatal intensive care unit [19–22]. Further, analyses of 97 devices used in the pediatric ICU found elution of multiple phthalates and alternative plasticizers [23]. Tri-octyl mellitate (TOTM), a close derivative of DEHP, is used increasingly as a substitute for DEHP in medical devices, particularly in Europe, due to rising concerns associated with the use of phthalate plasticizers. Infants undergoing cardiac surgery using TOTM-plasticized tubing had detectable levels of TOTM in the serum [19] and metabolites in their urine [24]. The amount of exposure can be significant. DEHP exposure was estimated to exceed the tolerable daily intake level of 50 μg/kg body weight per day more than 100-fold in neonates [25] and 37-fold in adults [17]. Collectively, these data indicate that phthalates in medical devices escape to contaminate the inpatient population.

Successful wound healing requires repair initiation and removal of debris by immune cells [26–28]. Damage associated molecular patterns (DAMPs) released by damaged cells are recognized by pattern recognition receptors on infiltrating monocytes and macrophages [29–32]. NLR family pyrin domain containing 3 (NLRP3) [29, 30] is the primary pattern recognition receptor activated by DAMPs [32]. *In vivo*, combined exposure to DEHP, BBzP and DIDP at doses comparable to levels experienced post-cardiac surgery activated the NLRP3 inflammasome, increased cardiac dilation and reduced cardiac systolic function, and increased the percentage of pro-inflammatory macrophages in hearts of a murine model of post-myocardial infarction (MI) [33]. Importantly, these effects were reduced by co-treatment with a NLRP3-specific antagonist. *In vitro*, DEHP, or its metabolite mono-ethylhexyl phthalate (MEHP), increased pro-inflammatory IL-1β and cytokines TNFα, IL-6 and IL-8 when added to human THP-1 or mouse RAW264.7 monocytes [34–36]. Together, these data imply that the acute phthalate exposure promotes excessive inflammasome activation and a pro-inflammatory monocyte and macrophage population.

DEHP is the most common plasticizer used in the manufacturing of plastics, including those in medical devices [37]. Its known toxicities highlight the need for safer alternatives [38]. Efforts have successfully resulted in the production of alternative plasticizers such as tung maleic triglycidyl ester [39]. Similarly, we, and others, have developed non-phthalate plasticizers based on maleate, succinate, and short linear diols. In addition to comparable or superior plasticizing ability compared to that of DEHP [40, 41], these non-phthalate-derived plasticizers are rapidly biodegraded to unstable and non-toxic metabolites in the environment [41]. Importantly, they can be synthesized from non-petroleum-derived chemical substrates [42] and demonstrate a safety profile in *in vitro* and *in vivo* toxicological studies [43–45]. These findings led us to test the hypothesis that non-toxic plasticizers have no or reduced inflammasome activation and are neutral on monocyte and macrophage wound infiltration. Further, in direct comparisons using testes-derived cell lines and testes organ cultures, succinate-based plasticizers were less toxic than MEHP, a DEHP metabolite, or DINCH, a commercial plasticizer [46]. Here, we report our findings based on measurements of inflammasome priming, innate immune cell infiltration and the impact on cardiac structure and function in male and female C57bl/6N mice exposed to DEHP, the emerging alternative plasticizer TOTM, and the non-phthalate succinate-based plasticizers, di-octyl succinate (DOS) and di-heptyl succinate (DHPS) during recovery from a surgically-induced MI. To identify activation of pattern recognition receptors in monocytes, we treated human monocyte THP-1 cells with the chemicals and quantified inflammasome expression and the downstream activation products caspase-1 and IL-1β.

## Methods

### Materials

DEHP (>99.5% pure, CAS 117-81-7), tri-octyl tri-mellitate (TOTM, > 99% pure, CAS 3319-31-1), and peanut oil (CAS 8002-03-7) were purchased from Sigma-Aldrich, (Oakville, ON). Di-octyl succinate (DOS) and di-heptyl succinate (DHPS) were synthesized as previously described [41, 42].

Nigericin (>98% pure, CAS 28643-80-3) was purchased from Cayman Chemicals (Ann Arbor, MI). Phorbol 12-myristate 13-acetate (PMA, >99% pure, CAS 16561-29-8) was purchased from Abcam, (Toronto, ON). Sulforhodamine B (SRB, (CAS 222-529-8, 85% pure) was purchased from Sigma-Aldrich (Oakville, ON). Antibodies specific for the active p20kD caspase-1 protein were purchased from Adipogen (San Diego, CA) and antibodies specific for IL-1β were purchased from Novus Biologicals (Toronto, ON) or GeneTex (Irvine, CA).

### *In vitro* monocyte cell culture and treatment of THP-1 cells

The male-human monocyte cell line, THP-1, was cultured in RPMI media containing 10% FBS and antibiotics. For experiments, 1.5 × 10^6^ cells / well were cultured in 6-well plates for 48 hours in media containing PMA, 50 ng/ml, to induce macrophage differentiation [47]. The media was then replaced to remove the PMA and 6 hours later, vehicle, chemicals at the concentrations indicated or the inflammation control LPS, 25ng/ml were added at the concentrations indicated. Chemicals were dissolved in absolute ethanol and serially diluted before use. Ethanol addition was similar regardless of concentration and did not exceed 0.4% of the media volume. Nigericin was dissolved in ethanol and 10 μM was added 20 hours after phthalate addition. Cell lysate or media were collected 24 hours after metabolite addition. Cell lysates were prepared for RNA extraction using GENEzol according to the instructions of the manufacturer (FroggaBio, Toronto, ON). Sulforhodamine staining was performed 24 hours after the addition of graded amounts of chemicals [48]. Shown are the representative results of independent experiments.

### Animal manipulation

All animal experiments were reviewed and approved before they began by the *Lady Davis Institute Animal Care Committee* according to the guidelines of the *Canadian Council on Animal Care*. Personnel in the Surgery Core who performed surgery are certified Animal Health Technicians with > 10 years of experience. All laboratory staff had successfully completed animal handling and surgical modules as stipulated by McGill University. C57bl/6N male and female mice, 3–6 months of age (Charles River, St. Constant, Que.) were fed a Harlan Teklad Global 2018 diet and acidified tap water, housed in polycarbonate cages with 1/4" corncob bedding and had a 12-hour dark/light schedule.

#### Myocardial infarction surgery and treatments post-MI

Before surgery, mice were randomly selected to the different treatment options. All surgery was performed by the Surgery Core of the *Lady Davis Institute* [49, 50]. Mice were treated with vehicle or test chemicals on each surgery day. Mice were anesthetized with 3% isoflurane 1.5L.min oxygen, anesthesia assured by the absence of a toe pinch reflex, intubated and placed on a heating pad. Ophthalmic ointment was applied. Mice were injected with slow-release buprenorphine, 1mg/kg body weight, to provide continual analgesia for the entire experimental time period of the next three days. Fur was removed from the chest with Nair. After Nair removal, the skin was washed and lidocaine–bupivacaine, 2mg/kg body weight, applied to provide surface analgesia. The chest was opened using a 1cm cut over the left thoracic area. Under a dissecting microscope, the muscles over the ribs were delicately separated and a retractor inserted to keep the area open The heart was exposed through a chest incision between the third and fourth ribs and the left anterior descending coronary artery approximately 1 mm distal to the left atrial appendage was ligated permanently using a 7–0 silk suture placing approximately 40% of the left ventricle at risk for infarction [51]. The muscles were placed back into position and the surface wound closed using 7–0 silk sutures. Animals recovered in a heated chamber and once awake and mobile were then returned to general housing. All animals recovered from surgery. All animals were monitored twice daily by Surgical Care staff and once daily by laboratory staff. Humane endpoints were determined by the Surgical Care staff with mice euthanized within one hour of notification. Eighty-eight mice were used in this study. One male mice was euthanized before experimental day 3 because it exceeded the pre-determined endpoints defined as piloerection, hunched posture and reduced or absent mobility. Six mice, 5 male and 1 female mouse, did not display humane endpoints and were found dead. Upon necropsy they showed evidence of cardiac rupture manifest as substantial clotted blood localized to the chest cavity. Despite monitoring these mice had not displayed humane endpoints symptoms because cardiac rupture is a sudden event.

The amount of DEHP, 25 mg/kg/day, is the mouse equivalent of human exposure detected 12-hours post-surgery [17]. The formula, human equivalent dose = [mouse equivalent dose × (metabolism constant (Km) for mice / Km for humans)] where a Km for mice of 3 and a Km for humans of 37 were used in this calculation [52]. TOTM, DOS and DHPS were delivered at a 200-fold lower dose than DEHP, 0.125mg/kg/day, to reflect their demonstrated and anticipated reduced leaching from plastic material. Chemicals in peanut oil or peanut oil alone were delivered orally for three days by a micropipettor.

#### Echocardiography

On day 3 post-MI, mice were anesthetized with isoflurane and echocardiography was performed using a VEVO 770 sonograph (VisualSonic, Toronto, ON). Left ventricle (LV) area and LV volume in systole and diastole, ejection fraction, cardiac output and stroke volume were calculated from EKV-gated long axis images using proprietary software as done previously [49, 50]. EKV-gated acquisitions of the short axis view at the level of the papillary muscles allowed calculation of the fractional area change (FAC) as ((LV area in diastole minus the LV area in systole) divided by the LV area in diastole) × 100% and is reported as a percentage.

#### Flow cytometry

Single cell suspensions of whole heart tissue were prepared as described previously [50]. Briefly, mice were euthanized on day 3 post-MI and the heart dissected into small pieces. After enzymatic digestion, cells were collected from the interface of a 40% / 80% Percoll gradient. Samples were incubated with Am-Cyan Live/Dead Fixable Dead Cell stain (Molecular Probes, Carlsbad, CA), blocked with 2.4G2 hybridoma (Fc Receptor Block, ATCC: HB-197), incubated with fluorescently labeled antibodies and fixed with 1% paraformaldehyde.

The following antibodies were used: Brilliant Violet 785-conjugated anti-CD45 (30-f11, BioLegend), Brilliant Violet 650-conjugated anti-MHCII (M5/114.15.2, BD Biosciences), phycoethythrin / Dazzle 594-conjugated CD64 (x54-5/7.1, BioLegend), efluor450-conjugated anti-CD11b (MI/70, eBioscience), Alexa 488-conjugated anti-Ly6G (RB6-8c5, eBioscience), APC-conjugated anti-Ly6C (AL-21, BD Biosciences), FITC-labelled anti-CD38 (90, eBioscience), and phycoethythrin-conjugated anti-MerTK (2b10c42, BioLegend). 123count e-beads (cat no. 01–1234, Affymetrix) were added before analyses. Flow cytometry used an LSR Fortessa Cell Analyzer (BD Biosciences, San Jose, CA) and DIVA software. Data were analyzed using FlowJo software v10.1 (Tree Star Inc., Ashland, OR).

#### Expression analyses

To quantify RNA expression changes, cultured cells or cardiac infarct tissue were homogenized in GENEzol and DNA-free RNA isolated according to the instructions of the manufacturer (FroggaBio, Toronto, ON.). Samples were prepared from mice which had surgery contemporaneously to avoid batch effects. First-strand cDNA was prepared using a SensiFast cDNA synthesis kit (cat. no. BIO-65053, FroggaBio, Toronto, ON). Quantitative PCR was performed using duplicates or triplicates, Green-2-Go qPCR Mastermix-Low ROX mix (cat. no. QPCR002-L, BioBasic Canada, Markham, ON), gene-specific primers and the Applied Biosystems 7500 Fast RT-PCR system (Life Technologies, Grand Island, NY). Primer sequences are shown in **S1 and S2 Tables**. Shown are data normalized to the housekeeping gene *hGADPH* for THP-1 cells and *m36B4* for mouse tissues. Fold change in gene expression was determined by the 2^−ΔΔCt^ method in comparison with expression in VEH treated samples.

Protein expression in cell culture media or cardiac infarct homogenates was measured by immunoblotting (IB). Infarct tissue was homogenized in RIPA buffer containing proteinase inhibitors (1% NP-40, 50 mM Tris (pH 7.4), 0.5% deoxycholate, 159 mM NaCl, 0.1% SDS, 10 mM sodium metabisulfite, proteinase inhibitor cocktail, (Roche, Indianapolis, IN), and 1mM PMSF), centrifuged briefly and the protein concentration in the supernatant quantified using a BCA assay (BioRad) according to the manufacturer’s instructions. Briefly, cell culture media (10 μL) or heart homogenate (20 μg) were separated using SDS-PAGE and electrophoretically transferred to Immobilon P membrane (Millipore, Bedford, MA). Membranes were stained with Ponceau S to verify equal protein loading, blocked, incubated overnight at 4°C with protein-specific antibodies, then with the species appropriate-secondary antibody complexed to horseradish peroxidase and the interaction revealed using chemiluminescent detection kits (Pierce Chemical Co., Rockford, IL). Several exposures were collected onto X-ray film. Companion membranes were permanently stained with Coomassie Brilliant Blue, destained and scanned using NIH Image J analysis software (NIH, Rockville, MD).

### Statistical analyses

Normality was verified by the Kolmogorov-Smirnov test. Differences between exposure groups as was evaluated by student-t-test for pairwise comparisons and one-way ANOVA or two-way ANOVA as appropriate for groups of > 2 using the statistical program SigmaStat 3.1 and the Tukey-Kramer post-hoc test. A p-value of < 0.05 was considered significant. Shown are the results of three independent THP-1 experiments. Cell survival and qPCR was performed in triplicates. Mice used in the study are: Males; VEH, n = 36, DEHP, n = 39, TOTM n = 14, DOS, n = 19, DHPS, n = 25 and Females; VEH, n = 25, DEHP, n = 20, TOTM, n = 14, DOS, n = 13, DHPS, n = 18. The numbers of mice used for each parameter are indicated in the figure legends.

## Results

### Response of macrophages to plasticizer exposure *in vitro*

**Fig 1:**
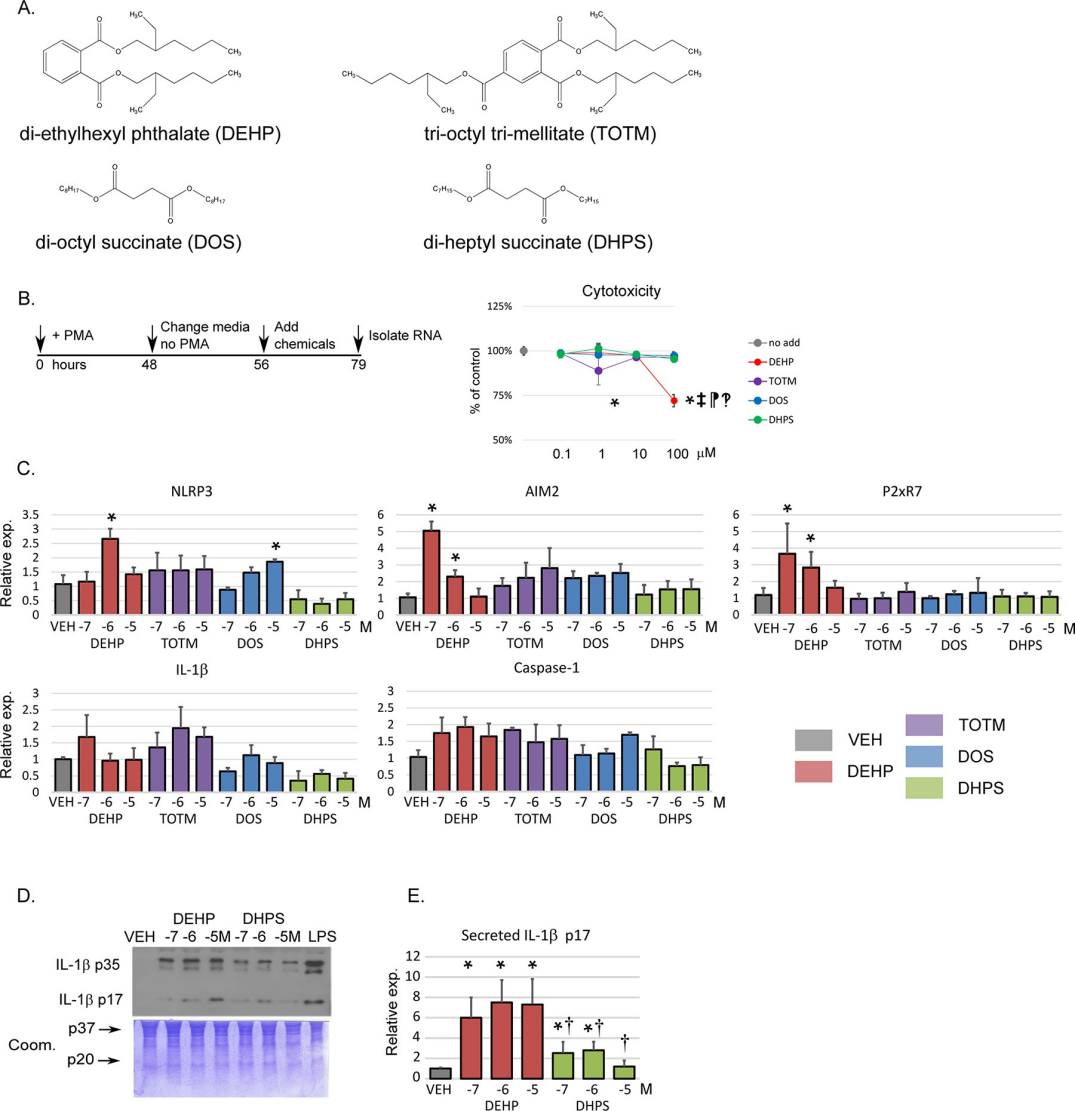
Impact of phthalate metabolites on macrophages *in vitro*. **A.** Compounds investigated in this study. **B.** Schematic of THP-1 differentiation and the timeline for chemical addition. THP-1 monocytes were differentiated into macrophages and then treated for 24 hours with ethanol (VEH) or the indicated chemicals. Chemicals were added in triplicate to cells to achieve 0.1, 1, 10 and 100 μM as the final concentration and the cells incubated for another 24 hours. Cells were fixed, stained with SRB and absorbance analyzed. Vehicle addition was arbitrarily indicated as 100%. Data are the mean ± SEM. Shown is the result of two independent experiments. A p-value of < 0.05 was considered significant and is indicated by an * in comparison with VEH, ‡ in comparison with DEHP, ¶ in comparison with DOS and? in comparison with DHPS. **C**. RNA was isolated 24 hours after chemical addition at 10^-7^M, 10^-6^M and 10^-5^M and Q-PCR was performed in duplicate using the indicated gene-specific primers and *hGAPDH*. The bar graphs are representative of three independent experiments. Expression of Vehicle treated cells was equal to 1.0. Data are the mean ± SEM. A p-value of < 0.05 was considered significant and is indicated by an * in comparison with VEH. **D**. Media from THP-1 cells treated with VEH, DEHP or DHPS at 10^-7^M, 10^-6^M and 10^-5^M concentration or the inflammation positive control LPS, (25ng/ml) was collected 24 hours after chemical addition at 10^-7^M, 10^-6^M and 10^-5^M. The media, 10 μl, was immunoblotted using anti-p17 kD IL-1β specific antibody. Shown are representative blots and Coomassie stained membranes of three independent experiments. **E.** The bar graph indicate the relative accumulation of p17 kD IL-1β into the media. Expression of Vehicle treated cells was equal to 1.0. A p-value of < 0.05 was considered significant and is indicated by an * in comparison with VEH and a † in comparison with the same concentration of DEHP.

To test the impact of conventional and new plasticizers *in vitro*, we differentiated human monocyte THP-1 cells to macrophages with PMA [47], then added DEHP, TOTM, DOS or DHPS and analyzed cytotoxicity, inflammasome expression and secretion of Caspase-1 and IL-1β, as seen in **Fig 1**. The chemical schematic and chemical addition timeline are shown in **Fig 1A and 1B**. The doses chosen, 0.1 and 1 μM, mimic DEHP levels often detected in the general population [16, 53]. The higher doses reflect exposure in post-cardiac surgery patients.

Cytotoxicity was only evident at the 100 μM dose of DEHP. No significant differences were detected for the addition of TOTM, DOS or DHPS at doses up to 100 μM, as seen in **Fig 1B**. To determine if chemical addition at concentrations ranging from 10^-7^M to 10^-5^M induced greater inflammasome receptor expression, we quantified RNA expression of the inflammasome receptors NLRP3, Absent-in-melanoma-2 (AIM2) and Purinergic receptor P2x ligand-gated ion channel 7 (P2xR7). DEHP treatment increased expression of NLRP3, AIM2 and P2xR7, as seen in **Fig 1C**. The addition of TOTM, DOS and DHPS had no effect. Caspase-1 and IL-1β are the effectors of NLRP3 activation. None of the chemicals modified expression of Caspase-1 or IL-1β. To assess whether chemical exposure increased active pro-inflammatory p17 kD IL-1β accumulation in the cell media, we treated PMA-stimulated THP-1 cells with increasing amounts of DEHP or DHPS and quantified protein expression in conditioned media, as seen in **Fig 1D**. Greater amounts of pro-inflammatory p17 kD IL-1β protein were found in the media of DEHP exposed versus DHPS exposed cells. Our *in vitro* data suggest that treatment of macrophages with DEHP induced inflammasome priming and secretion of the pro-inflammatory mediator IL-1β. In contrast, fewer changes were noted in cells treated with TOTM or the novel non-phthalate plasticizers DOS or DHPS.

### Impact of *in vivo* exposure in male and female mice to phthalates post-MI

**Fig 2:**
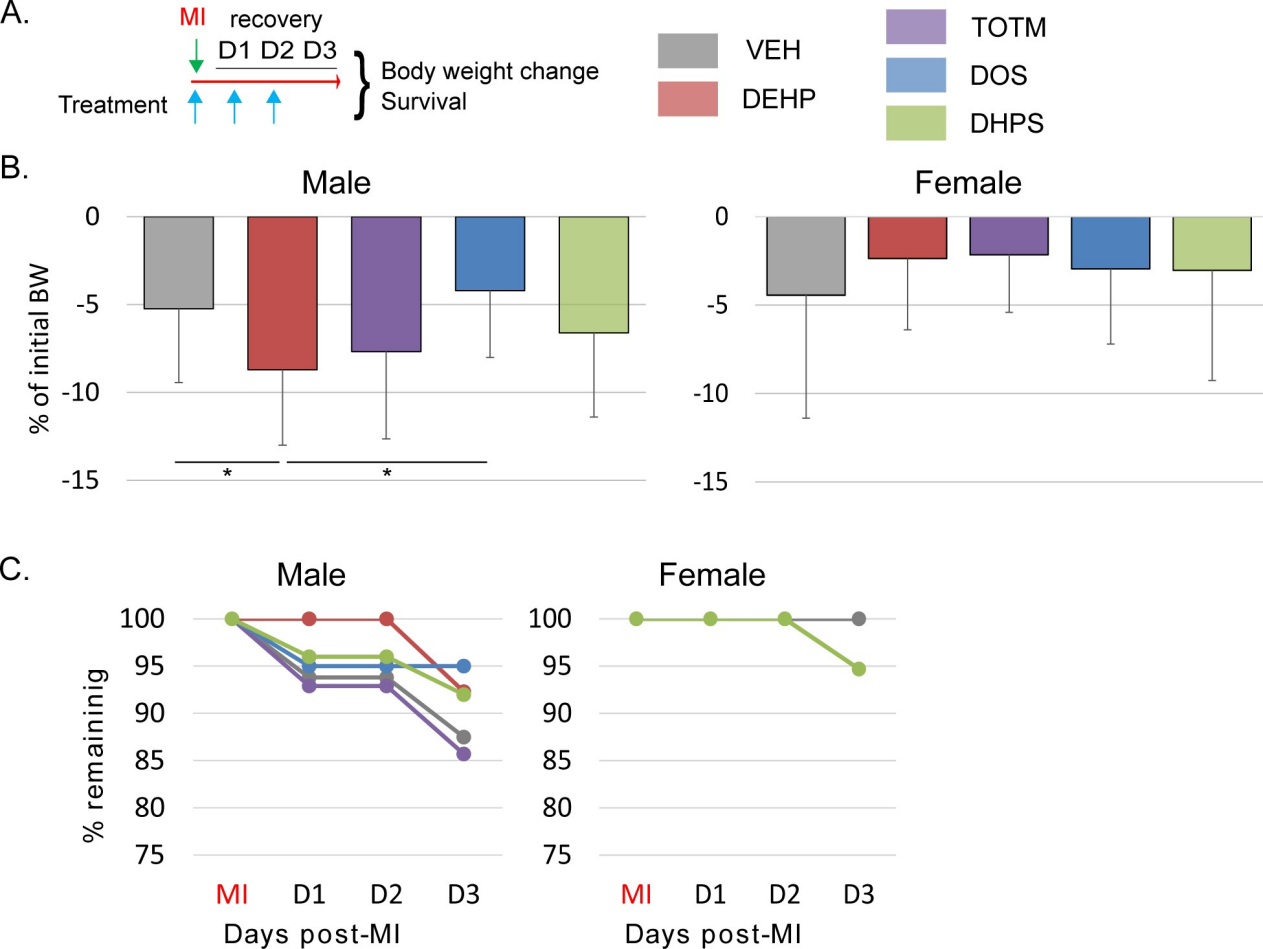
Impact on body weight and recovery. **A**. Timeline of chemical treatment relative to surgery. Surgery to create a myocardial infarction was performed on male and female C57bl/6N mice as described. Mice were randomly assigned to one of five groups before surgery. All chemicals were diluted in peanut oil. *Group 1*. **Vehicle**, peanut oil. *Group 2*. **DEHP**, 25 mg/kg/day. *Group 3*. **TOTM**, 0.125 mg/kg/day. *Group 4*. **DOS**, 0.125 mg/kg/day. *Group 5*, **DHPS**, 0.125 mg/kg/day. Males; VEH, n = 36, DEHP, n = 39, TOTM n = 14, DOS, n = 19, DHPS, n = 25. Females; VEH, n = 25, DEHP, n = 20, TOTM, n = 14, DOS, n = 13, DHPS, n = 18. Treatments were repeated daily until euthanasia on day 3 post-surgery. **B.** Percentage body weight change. The body weight was measured on the day of MI surgery and upon euthanasia on Day 3. The percentage of body weight lost after surgery was calculated. Significance is indicated by an * in comparison with VEH treated mice. **C.** Percent recovery. The number of mice remaining after surgery was monitored daily. Mice were euthanized and counted as not-alive if moribund.

Tubing plasticized by DEHP eluted 1/8 of the incorporated DEHP within 24 hours of testing. In comparison, TOTM elution was 1/180^th^ of the incorporated material after 24 hours in the ethanol solution, ~1/116^th^ in the chloroform solvent [54, 55], and was 350-fold lower in sheep blood [56], 200-fold lower from phospholipid coated tubing [57], and 270-fold less into nutritional and pharmaceutical solutions than DEHP [58]. Here, we tested the impact of 200-fold lower exposure of TOTM, DOS and DHPS (0.125mg/kg/day) relative to DEHP (25mg/kg/day) to inform on the impact of amounts anticipated in the post-surgical population. To evaluate the impact of plasticizers with sex *in vivo*, we treated male and female mice with DEHP, TOTM, DOS, and DHPS during recovery from an experimentally induced MI. The surgery timeline and chemical treatment for all *in vivo* experiments is shown in **Fig 2A.** All mice lost body weight after surgery. Males treated with DEHP and TOTM lost a greater percentage of body weight compared to that of VEH or DOS treated mice, as seen in **Fig 2B**. Treatments did not influence body weight loss in similarly treated female mice. Male mice tended to show reduced survival irrespective of treatment, as seen in **Fig 2C**. In females, only DHPS treated mice showed some reduced survival.

**Fig 3:**
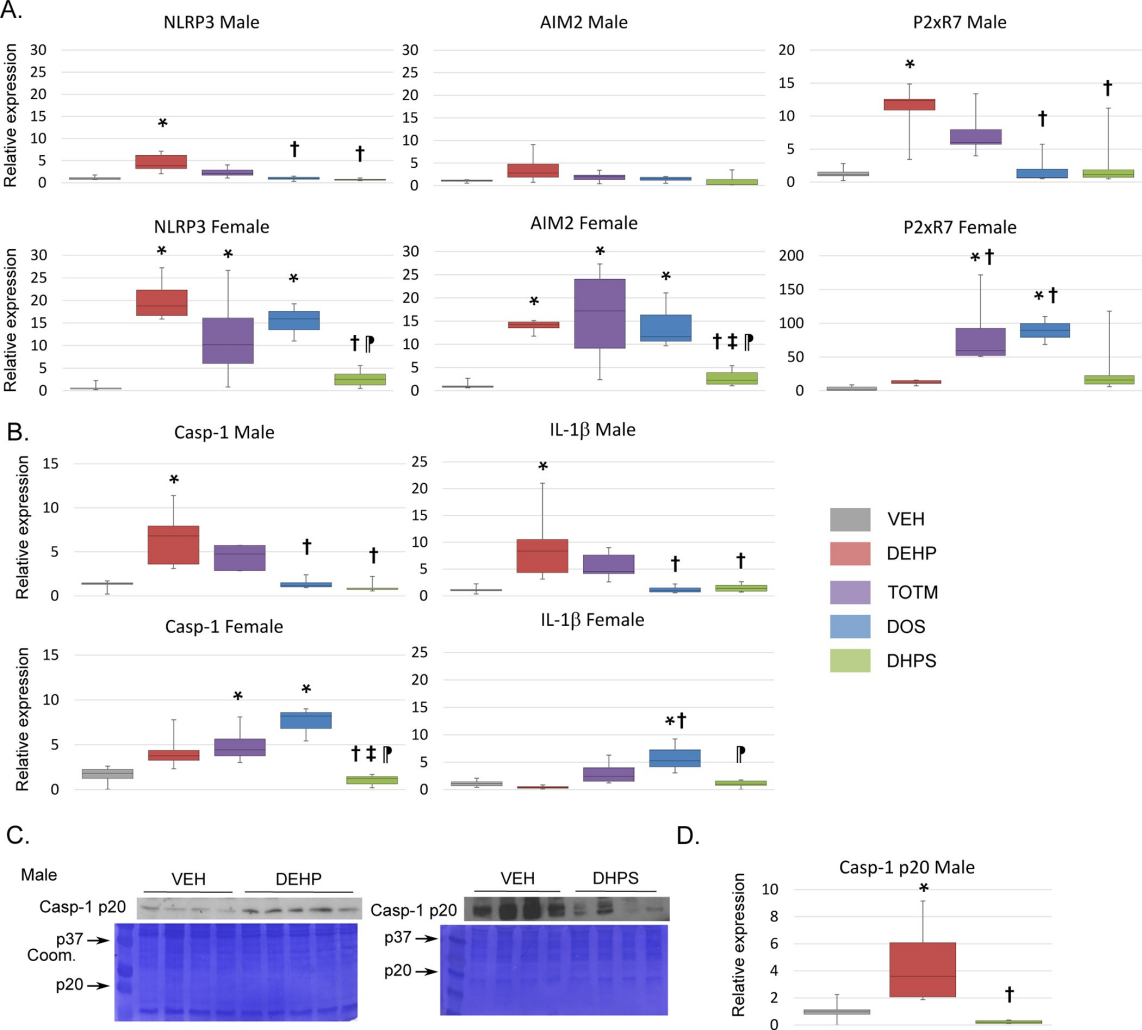
Influence of chemical exposure on expression of inflammasome related genes. **A and B.** RNA expression in the infarct. RNA was isolated from the heart infarcts of male and female mice and Q-PCR performed using primers specific for *m36B4* and the indicated genes. Males; VEH n = 5, DEHP n = 6, TOTM n = 5, DOS n = 4 and DHPS n = 5. Females; VEH n = 6, DEHP n = 5, TOTM n = 5, DOS n = 3 and DHPS n = 6. The box plots show the combined results. Expression in the VEH samples was artificially designated as 1.0. The median and minimum and maximum values are displayed in the box plots. A p-value of < 0.05 was considered significant and is indicated by an * in comparison with VEH, † in comparison DEHP, ‡ in comparison with TOTM and ⁋ in comparison with DOS. **C and D.** Caspase-1 p20 protein expression in the infarct. Protein was isolated from heart infarct samples collected from VEH n = 4, DEHP n = 4 and DHPS n = 4 exposed male mice and immunoblotted using anti-p20 kD Caspase-1 specific antibody. Shown are representative blots and membranes stained with Coomassie Blue. Expression in the VEH samples was artificially designated as 1.0. The median and minimum and maximum values are displayed in the box plots. A p-value of < 0.05 was considered significant and is indicated by an * in comparison with VEH and †in comparison DEHP.

Next, we isolated mRNA from cardiac infarcts and used Q-PCR to quantify the expression of the pattern recognition receptors NLRP3, AIM2 and P2xR7, as seen in **Fig 3A**. In males, DEHP exposure increased expression of NLRP3 and P2xR7 when compared to VEH, DHPS or DOS groups. AIM2 expression was unaffected by plasticizer exposure. For female mice, DEHP, TOTM and DOS increased NLRP3 and AIM2 expression. TOTM and DOS increased AIM2 and P2xR7 expression. NLRP3, AIM2 and P2xR7 expression was similar in VEH and DHPS treated male and female mice. In males, DEHP exposure increased Caspase-1 and IL-1β expression, as seen in **Fig 3B**. In females, TOTM increased Caspase-1 expression, and DOS exposure increased Caspase-1 and IL-1β expression. No increases were detected in male or female DHPS treated mice. We performed immunoblots to evaluate whether mRNA expression affected protein translation in DEHP and DHPS exposed male mice. Males treated with DEHP, but not DHPS, increased expression of the active p20 kD Caspase-1 protein, as seen in **Fig 3C and 3D**. We conclude that exposure to DEHP, TOTM and DOS increased mRNA expression and thereby priming of the NLRP3 inflammasome. In contrast, exposure to DHPS had no effect.

### Impact on cardiac structure / function

**Fig 4:**
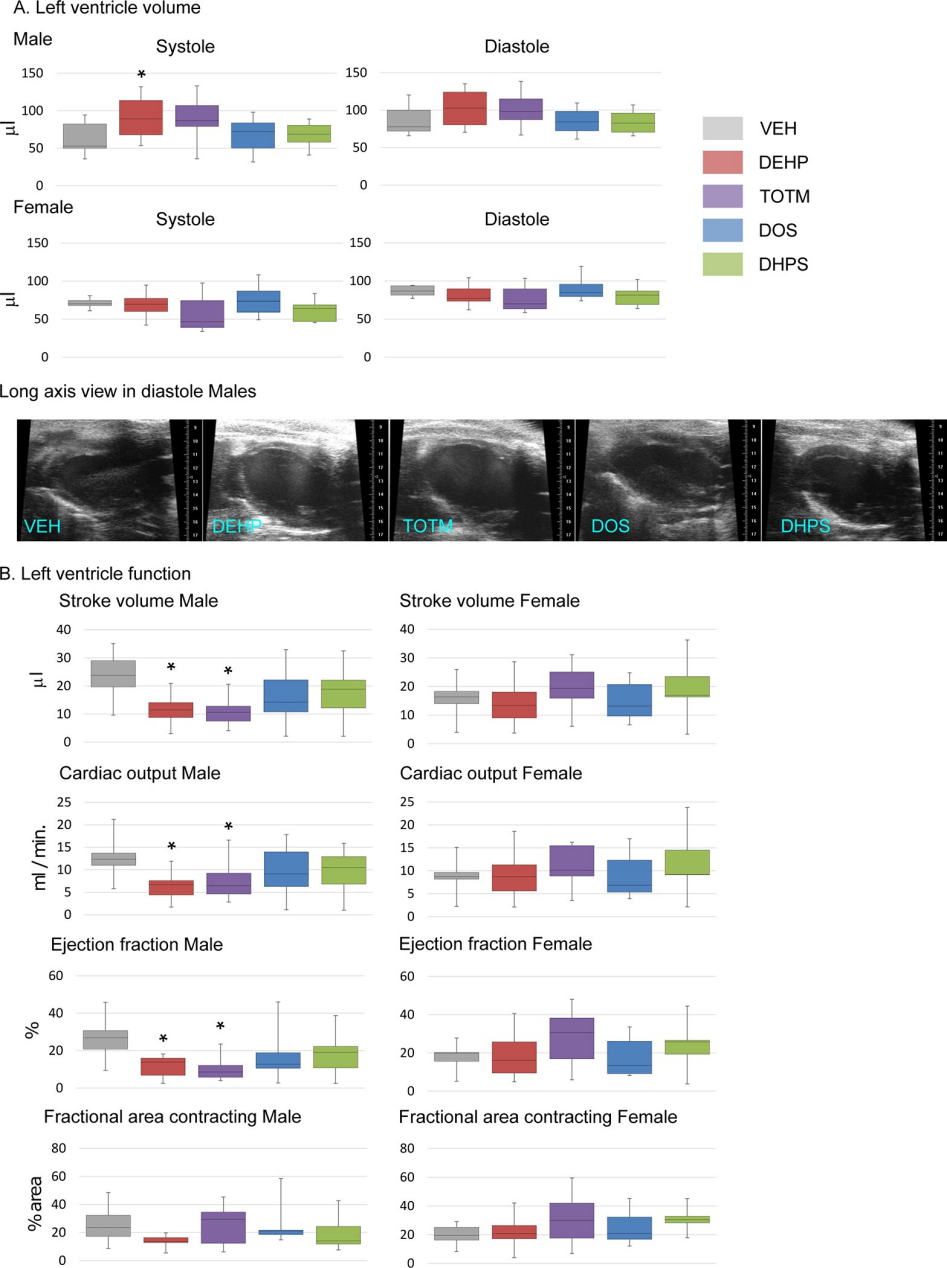
Influence of acute exposure on cardiac structure/function remodeling after an MI. **A. Left ventricle volume.** Echocardiography acquisitions were obtained for male and female mice on Day 3 post-MI. The LV internal volume in systole and diastole were calculated using the long axis view and EKV-gated acquisitions. The box plots represent the results of Male; VEH n = 12, DEHP n = 10, TOTM n = 9, DOS n = 9 and DHPS n = 12 and Female; VEH n = 10, DEHP n = 13, TOTM n = 8, DOS n = 8 and DHPS n = 9 mice. Shown are the median and minimum and maximum values. Representative sonographic images captured from the long axis view are shown. **B. Left ventricle function.** The LV stroke volume, cardiac output, ejection fraction and fractional area change were calculated using the long and short axis views and EKV-gated acquisitions. The box plots represent the median and minimum and maximum values for male and female mice. A p-value of < 0.05 was considered significant and is indicated by an * in comparison with VEH, †in comparison DEHP, ‡ in comparison with TOTM and ⁋ in comparison with DOS.

To determine if chemical exposure modified cardiac structure and function after an MI, we performed echocardiography on Day 3 post-MI, as seen in **Fig 4A**. The LV volume was increased in male mice exposed to DEHP. Calculation of echocardiography-derived LV systolic function measurements identified reduced stroke volume, cardiac output and ejection fraction in male mice treated with DEHP and TOTM, as seen in **Fig 4B**. In contrast, LV volume and LV systolic function in females was unaffected by plasticizer exposure. There was no change in males or females in the fractional area contracting, regardless of treatment. In summary, DEHP and TOTM exposure negatively influenced cardiac and function structure in male, but not female mice. Further, neither DOS nor DHPS exposure had no impact cardiac structure and function, regardless of sex.

### Immune cell infiltration into cardiac tissue post-MI

**Fig 5:**
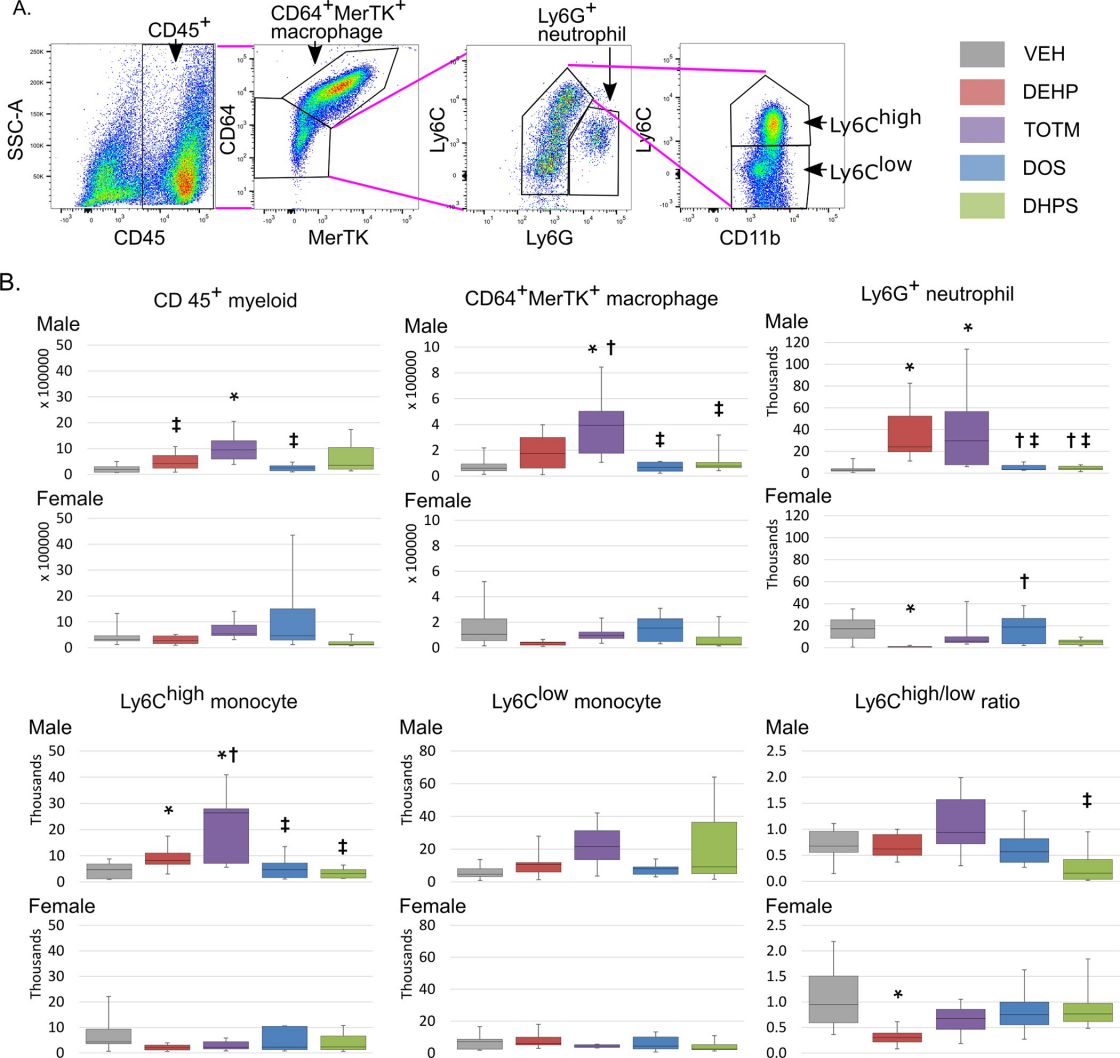
Characterization of the immune cell infiltration into the entire heart. **A. Gating strategy.** The gating strategy and markers to identify cell types is shown. Live cells were gated for CD45 expression. Macrophages (CD64^+^MerTK^+^), neutrophils (CD11b^+^Ly6G^+^) and monocytes (CD11b^+^Ly6G^low^) were identified. Monocytes were then sub-classified into CD11b^+^Ly6C^high^, and CD11b^+^Ly6C^low^. **B. Flow cytometry of single cell preparations isolated from whole hearts post-MI.** Single cell preparations were prepared from the entire heart on day 3 post-MI. The number and identification of the indicated cell types present is indicated. Male; VEH n = 10, DEHP n = 10, TOTM n = 9, DOS n = 7 and DHPS n = 9 and Female; VEH n = 9, DEHP n = 10, TOTM n = 7, DOS n = 8 and DHPS n = 7 mice. The box plots represent the median and minimum and maximum values of each group. A p-value of < 0.05 was considered significant and is indicated by an * in comparison with VEH, †in comparison DEHP and a ‡ in comparison with TOTM.

NLRP3 inflammasome activation and increased cardiac dilation coupled with reduced cardiac function are associated with increased innate immune cell infiltration in damaged tissues. Thus, we quantified the innate immune cells in whole heart tissue by flow cytometry, as seen in **Fig 5A and 5B**. We detected an increase in CD45^+^ myeloid cells in male mice exposed to TOTM versus VEH, as seen in **Fig 5B**, and when compared to DEHP or DOS exposed mice. Female mice demonstrated no difference in CD45^+^ myeloid cell infiltration, regardless of treatment. To discriminate the cell types involved, we sub-fractionated the myeloid cells. In male mice, DEHP exposure increased CD11b^+^Ly6G^+^ neutrophil and CD11b^+^Ly6C^high^ monocytes. Also, in male mice, TOTM exposure increased CD11b^+^Ly6G^+^ neutrophil, CD64^+^MerTK^+^ macrophage and CD11b^+^Ly6C^high^ monocyte accumulation. In females, DEHP reduced CD11b^+^Ly6G^+^ neutrophil accumulation. Exposure to DOS or DHPS had no effect on myeloid subtype accumulation. Although neither CD11b^+^Ly6C^high^ nor CD11b^+^Ly6C^low^ monocyte numbers were affected individually, the ratio of accumulation CD11b^+^Ly6C^high^ / CD11b^+^Ly6C^low^ monocyte was reduced in DEHP-exposed female mice. We conclude that DEHP and TOTM exposure increased innate immune cell infiltration into heart tissue post-MI, with the greatest effect observed in male mice.

**Fig 6:**
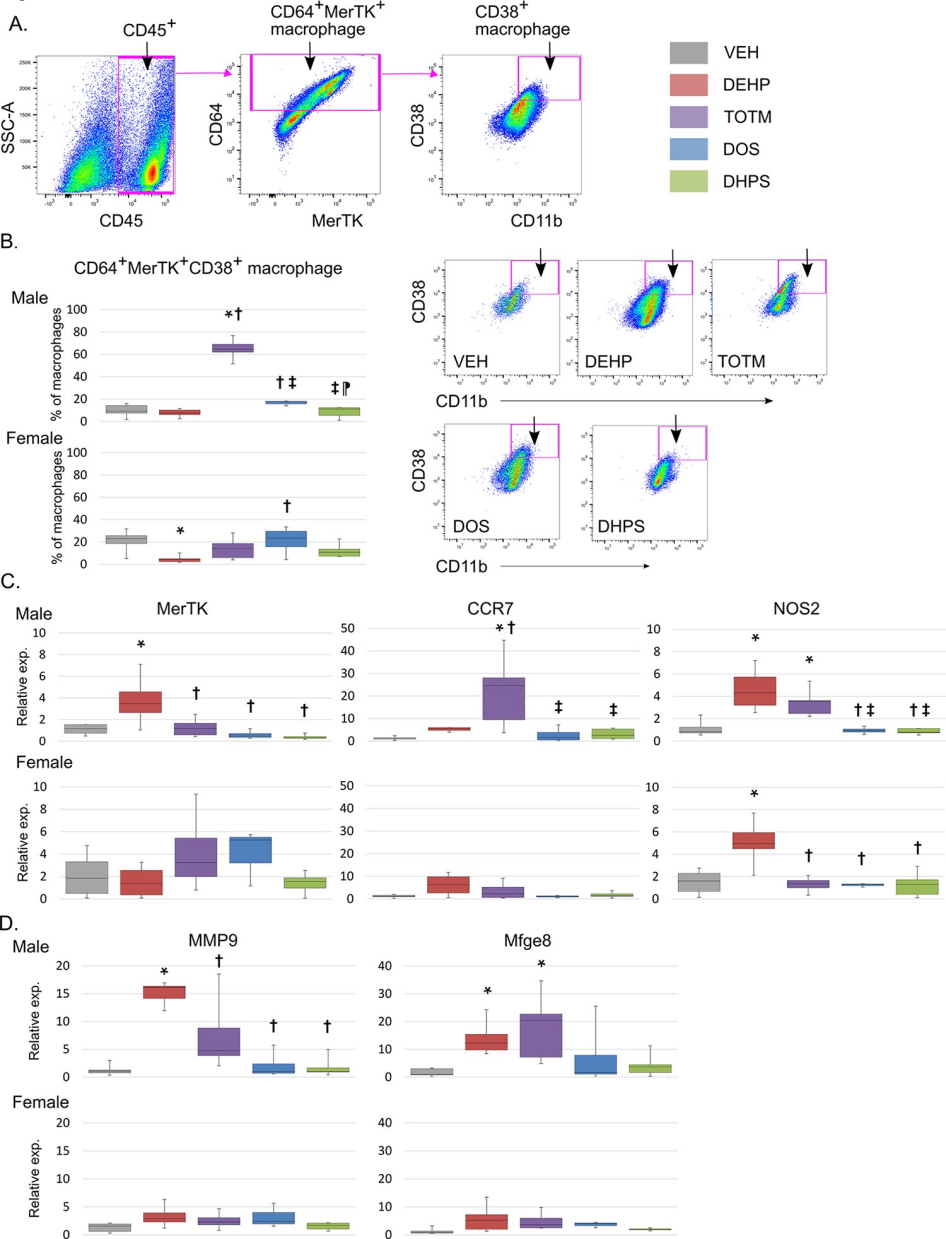
Subclassification of CD64^+^MerTK^+^ macrophages. **A. Gating strategy.** The gating strategy and markers to identify CD38^+^ macrophages is shown. CD64^+^MerTK^+^ macrophages were separated into those co-expressing CD38 and the percentage co-expressing the three proteins relative to the number of CD64^+^MerTK^+^ cells calculated. **B. Flow cytometry.** Male; VEH n = 10, DEHP n = 10, TOTM n = 9, DOS n = 7 and DHPS n = 9 and Female; VEH n = 9, DEHP n = 10, TOTM n = 7, DOS n = 8 and DHPS n = 7 mice. The box plots represent the median and minimum and maximum values of each group. A p-value of < 0.05 was considered significant and is indicated by an * in comparison with VEH, †in comparison DEHP and a ‡ in comparison with TOTM. Representative images of gated CD64^+^MerTK^+^CD38^+^ macrophages are shown. **C. RNA expression of macrophage-related genes in the infarct.** RNA was isolated from infarcts of mice and Q-PCR performed using primers specific for *m36B4* and the indicated macrophage genes. Male; VEH n = 5, DEHP n = 4, TOTM n = 5, DOS n = 4 and DHPS n = 5 and Female; VEH n = 6, DEHP n = 5, TOTM n = 4, DOS n = 3 and DHPS n = 6 mice. The box plots represent the median and minimum and maximum values of each group. Expression in the VEH samples was artificially designated as 1.0. A p-value of < 0.05 was considered significant and is indicated by an * in comparison with VEH, †in comparison DEHP, ‡ in comparison with TOTM and ⁋ in comparison with DOS.

We used the pro-inflammatory macrophage marker, CD38, to subcategorized CD64^+^MerTK^+^ macrophages, **Fig 6A and 6B**. In males, TOTM-exposure increased the percentage of CD64^+^MerTK^+^ macrophages co-expressing CD38 whereas similarly exposed females displayed no change. Moreover, DEHP-treatment in females reduced the percentage of CD38^+^CD64^+^MerTK^+^ macrophages. DOS and DHPS did not impact the percentage of CD38^+^CD64^+^MerTK^+^ macrophages when compared with VEH mice.

To assess pro-inflammatory monocytes / macrophages markers and macrophage function within the infarct, we isolated RNA from hearts and performed Q-PCR, as seen in **Fig 6C and 6D**. In DEHP treated males, as expected we detected increased expression of the monocyte / macrophage marker MerTK but also C-C chemokine receptor type 7 (CCR7), the pro-inflammatory marker nitric oxide synthase-2 (NOS2), the functional proteins matrix metalloproteinase-9 (MMP9), and the phagocyte-associated marker milk fat globule-EGF factor protein-8 (Mfge8). In TOTM treated males, we detected increased expression of CCR7, NOS2 and Mfge8, but not MERTK. In contrast, NOS2 expression was increased in DEHP-treated female mice.

Together, these data suggest that increased numbers of pro-inflammatory macrophages lie within the infarcts of male mice treated with DEHP or TOTM and that this phenotype is absent in males and females treated with DOS or DHPS.

## Discussion

Multiple plasticizers are incorporated into the plastics found in medical devices [23, 55, 59, 60]. The murine DEHP exposure modeled here is based on the amount of DEHP detected in adult cardiac patients 24 hours post-surgery [17]. This high-dose and acute exposure to DEHP impaired recovery of damaged cardiac tissue which was most evident in males. In male mice, DEHP exposure increased: (1) body weight loss; (2) NLRP3 and P2xR7 priming and active caspase-1 protein; (3) cardiac dilation; and (4) macrophage and neutrophil infiltration in the damaged tissue. In contrast, DEHP exposure in females led to (1) less body weight loss; (2) no increase in cardiac dilation, and (3) no elevation in innate immune cell infiltration in the damaged heart. Importantly, whereas cardiac systolic function was reduced in males a similar reduction was not detected in females. Like DEHP, TOTM exposure increased the percentage of pro-inflammatory macrophages in the infarct. Importantly, recovery of mice exposed to DOS or DHPS resembled VEH exposed mice. Overall, these data suggest that DEHP and TOTM impair healing post-MI and supports further investigation for the utility of non-phthalate plasticizers such as DOS and DHPS.

Patients developing weight loss of >5% [61] and LV dysfunction after an MI are at a high risk for a number of adverse outcomes, including recurrent MI, sudden cardiac death, and heart failure [62]. In addition to increased weight loss, we observed that male mice exposed to DEHP and TOTM exposure had a tendency towards greater cardiac dilation and significantly reduced cardiovascular systolic function when compared to VEH, DOS, or DHPS exposed males. In contrast, female mice experienced no changes in weight, cardiac structure, or cardiac function. These results indicate that DEHP and TOTM exposure is more consequential in males, disrupting LV geometric remodeling and LV function. Consistent with our results, DEHP exposure has been observed to lead to disrupted autonomic regulation, heart rate variability, and cardiovascular reactivity [63]. Similarly, phthalate exposure increased atrioventricular node and ventricular effective refractory periods, indicating disrupted cardiac electrophysiology [64] and induced a greater risk for cardiometabolic disease [65–67]. A retrospective study analyzing urine DEHP and metabolite levels in samples collected during NHANES 2001–2010 with mortality of the men and women up to 2015, found an increased risk for cardiovascular mortality in men with higher exposure [68]. Together, these data imply that DEHP and TOTM exposure promotes cardiovascular dysfunction and worsens dysfunction after a cardiac injury. Importantly, mice exposed to DOS and DHPS did not develop these cardiac function changes supporting their potential as less hazardous plasticizers.

Increased DEHP exposure after tissue damage impairs recovery. DEHP-driven inflammasome activation and increased pro-inflammatory IL-1β secretion from infiltrating macrophages may be part of the mechanism for greater tissue damage post-MI with some selectivity for males. Regulation of mRNA expression is the crucial inflammasome-controlling step [69] and, mechanistically, increases in IL-1β have the potential to bind to IL-1R expressed by neighboring cardiac cells [29, 30, 70] thereby propagating and enhancing the inflammasome response. The increased expression of NLRP3, AIM2 and P2xR7 receptors and Caspase-1 effector in DEHP-exposed mice suggest increased priming. Increased NLRP3, AIM and P2xR7 expression and increased secretion of active p20kD Caspase-1 and p17kD IL-1β was found in THP-1 monocytes exposed to doses of DEHP expected post-surgery. Consistently, DEHP, its metabolite MEHP and di-butyl phthalate have activated NLRP3 and increased Il-1β secretion in human and rat monocyte cell lines [33–36] as well as other cell lines [71–74]. In contrast, increases in pattern recognition receptor or effector expression were not detected with TOTM, DOS or DHPS exposure in THP-1 cells suggesting that these chemicals do not induce substantial inflammasome activation in macrophages. Together, these data suggest that inflammasome priming may not be a conserved feature of plasticizers. Recently, male-specific increases in NLRP3 at a surgical site and pain-like behaviors were identified in mice [75]. Similar to the results here, increased NLRP3 and IL-1β mRNA expression was detected in the injured male mice when compared with injured female mice. In a global brain-ischemia model, expression of NLRP3, ASC, caspase1 and IL-1β mRNAs were increased in the ischemic brain area within the first 3 days of injury in ovariectomized rats but were reduced in rats which had estrogen supplementation [76]. Additionally, men with aortic aneurysm and cancer had greater increased mRNA levels of NRLP3 than women [77, 78]. These data suggest that estrogen suppresses NLRP3 expression. The impact of sex on NLRP3 expression and activation may be model and tissue dependent. In the kidney, concomitant Caspase-1 deficiency in *Pkd1*^*RC/RC*^ mice genetically predisposed to developing cystic disease protected the female but not male mice [79]. These data suggest immune-mediated mechanisms underlie postoperative responses, are sex-specific and influenced by NLRP3 inflammasome assembly. In support of this notion, emerging evidence has shown that estrogen can act as an inflammatory protective factor to suppress NRLP3-mediated inflammation [76, 80].

Plasticizers have chemical-specific impacts on macrophage infiltration. In a previous study, we observed that combined exposure to DEHP and bisphenol A elevated monocyte, CD64^+^MerTK^+^ macrophage, and neutrophil infiltration post-surgery in male mice [17]. Here, DEHP-alone did not increase CD64^+^MerTK^+^ macrophages or alter the percentage of CD38^+^ macrophages. However, TOTM-exposure induced increased macrophage numbers and a greater percentage of pro-inflammatory CD64^+^MerTK^+^CD38^+^ macrophage accumulation in the heart. Additionally, TOTM-exposure coincided with greater expression of M1-type markers, CCR7 and NOS2 as well as MMP9 and Mfge8, markers of increased motility and phagocytosis. In other studies, MEHP treatment increased CCL2 expression, and neutrophil and macrophage infiltration in testis [81, 82]. These data suggest that TOTM induces an activated and pro-inflammatory macrophage cell population in the damaged tissue. In contrast, exposure to DOS and DHPS did not lead to increased macrophage numbers, and no increase in the percentage of infiltrating pro-inflammatory macrophages. Consistent with the lack of any change in body weight and echocardiography data in females, plasticizers had no influence on immune cell infiltration in female mice.

### Relevance

Patients may experience delayed recovery after cardiac surgery [83]. We and others have found increased urine levels of DEHP and other plasticizer metabolites after cardiac interventions [15–20, 22] with lower cytokine levels and reduced inflammation linked with surgeries with fewer devices [84, 85]. These data suggest that plasticizers leaching from medical devices may influence recovery by increasing the pro-inflammatory response. Our study suggests that testing of plasticizers and their alternatives involve *in vivo* models which simulate their use in the clinical setting.

Mice exposed to the succinate-based plasticizers DOS and DHPS during recovery from an MI resembled the recovery of VEH-treated animals. Comparing the two succinate-derived plasticizers, DHPS induced fewer changes than DOS. Taken together, these succinate-based plasticizers are promising candidates for the replacement of phthalate-based plasticizers. In addition to their performance in the context of a surgical recovery, these bio-based plasticizers are environmentally appealing because their manufacturing components are extracted from renewable resources. It should be noted that a complete life-cycle analysis is needed to conclusively show such plasticizers are concretely better than existing ones. In consideration of the aforementioned data, we rank the safety of plasticizers from safest to most toxic in the following order: DHPS > DOS >>> TOTM > DEHP.

### Limitations

We acknowledge several limitations to this study. Migration date is sparse for the majority of alternative plasticizers, including DOS and DHPS. The limited data available showed undetectable leaching of DOS and DHPS from PVC into water after one-week and three-week incubations [86]. However, release of DHPS into hexanes after 4 hours and 50°C, and vegetable oil after 2 and 4 weeks incubation was greater than DEHP [87]. Leaching of DOS or DHPS from PVC blends into sheep blood or mouse blood, urine or tissues after *in vivo* exposure have not been reported. Further, we cannot predict responses to lower or higher exposures. Surgical patients are exposed to plasticizers via many devices and multiple routes. Chemical delivery here does not replicate the multiple routes for exposure such as via IV, dermatological and indwelling devices as well as exposure through the surgical drapes and gloved used by surgeons and nurses. Chemical exposure levels were not measured in urine or serum samples. We sought to examine the near surgery impact of chemical exposure and did not examine recovery of the mice beyond Day 3 post-MI. Hence, we cannot comment on whether increased exposure would delay or enhance long-term healing. Further investigation including use of older aged mice, examination of longer recovery times and the possibility that a poor diet may influence recovery post-surgery are necessary and critical to better understand how plasticizers leaching from medical devices influences recovery and to determine if non-phthalate plasticizers would be non-toxic under these conditions.

## Supporting information

S1 Table Primers sequences used in Q-PCR for human THP-1 macrophages are listed. (DOCX)

S2 Table Primers sequences used in Q-PCR for mouse infarct samples are listed. (DOCX)

S1 Raw images (PDF)
