# Supplementary material for: Exposure to the non-phthalate plasticizer di-heptyl succinate is less disruptive to C57bl/6N mouse recovery from a myocardial infarction than DEHP, TOTM or related di-octyl succinate
Source: PLoS One. 2023 Jul 13;18(7):e0288491. doi: 10.1371/journal.pone.0288491 (PMC10343165; doi:10.1371/journal.pone.0288491)
Supplement: S1 Table — (DOCX) [file pone.0288491.s001.docx]

Supplemental Table 1. Human Q-PCR primers

| Gene | Forward (5’ to 3’) | Reverse (5’ to 3’) | Amplicon (bps) |
| --- | --- | --- | --- |
| GAPDH | GAAGGTGAAGGTCGGAGTC | GAAGATGGTGATGGGATTTC | 226 |
| NLRP3 | CCACAAGATCGTGAGAAAACCC | CGGTCCTATGTGCTCGTCA | 91 |
| AIM2 | AGCAAGATATTATCGGCACAGTG | GTTCAGCGGGACATTAACCTT | 97 |
| P2xR7 | TATGAGACGAACAAAGTCACTCG | GCAAAGCAAACGTAGGAAAAGAT | 95 |
| Caspase-1 | GGCTCAGAAGGGAATGTCAA | TCACCCCACTCTATCCTTG | 108 |
| IL-1β | CCACAGACCTTCCAGGAGAA | GTGATCGTACAGGTGCATCG | 121 |
