# Supplementary material for: Exposure to the non-phthalate plasticizer di-heptyl succinate is less disruptive to C57bl/6N mouse recovery from a myocardial infarction than DEHP, TOTM or related di-octyl succinate
Source: PLoS One. 2023 Jul 13;18(7):e0288491. doi: 10.1371/journal.pone.0288491 (PMC10343165; doi:10.1371/journal.pone.0288491)
Supplement: S2 Table — (DOCX) [file pone.0288491.s002.docx]

Supplemental Table 2. Mouse Q-PCR primers

| Gene | Forward (5’ to 3’) | Reverse (5’ to 3’) | Amplicon (bps) |
| --- | --- | --- | --- |
| 36B4 | GGCACCGAGGCAACAGTT | TCATCCAGCAGGTGTTTGACA | 138 |
| GAPDH | CATGGCCTTCCGTGTTCCTA | GCGGCACGTCAGATCCA | 55 |
| NLRP3 | CCACAAGATCGTGAGAAAACCC | CGGTCCTATGTGCTCGTCA | 91 |
| P2xR7 | TGTGAAGTCTCTGCCTGGTG | TGTCCCCTAGTCGGAAGATG | 217 |
| AIM2 | ACCCGCAGTGACAATGACTT | TGTTCTGCCACCATCTGTTT | 172 |
| Caspase-1 | GCCCAAGCTTGAAAGACAAG | GGCCTTCTTAATGCCATCAT | 152 |
| IL-1β | GAAATGCCACCTTTTGACAGTG | TGGATGCTCTCATCAGGACAG | 116 |
| MerTK | CAGGGCCTTTACCAGGGAGA | TGTGTGCTGGATGTGATCTTC | 111 |
| CCR7 | AACCAAAAGCACAGCCTTCC | ACGTTTTTCCTGGGTTTCCC | 171 |
| NOS2 | TTCACCCAGTTGTGCATCGACCTA | TCCATGGTCACCTCCAACACAAGA | 140 |
| MMP9 | TCACACGACATCTTCCAGTACC | ACCTCATGGTCCACCTTGTTC | 104 |
| Mfge8 | TTGGGAAGGCTGGATAATCAGG | GTGATGATTCCTGTCACTTGCC | 116 |
