# Supplementary material for: Exposure to the non-phthalate plasticizer di-heptyl succinate is less disruptive to C57bl/6N mouse recovery from a myocardial infarction than DEHP, TOTM or related di-octyl succinate
Source: PLoS One. 2023 Jul 13;18(7):e0288491. doi: 10.1371/journal.pone.0288491 (PMC10343165; doi:10.1371/journal.pone.0288491)

Supplementary information

These images were scanned as PDFs using a CanoScan 4100 F scanner and ArcSoft Photo Studio 5.0 software.
The images were imported into Inkscape 0.92.4 and cropped from the entire film. The pink boxes outline the areas used in the figures.

Original scans of immunoblots - THP-1 Figure 1

Il-1 beta, E62 Thp-1 media

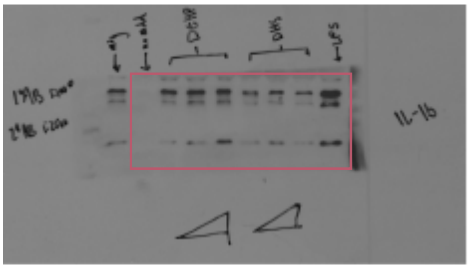

Caspase -1, E62 Thp-1 media

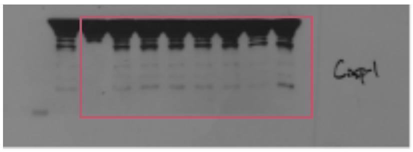

Original scans of immunoblots - mice Figure 3

Male, VEH vs. DEHP Caspase-1

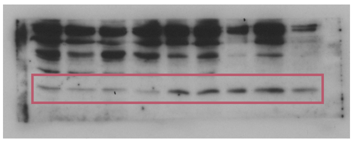

p37 →
p20 →

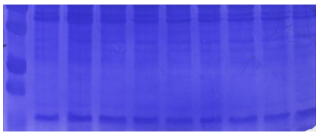

Male, VEH vs. DHPS Caspase-1

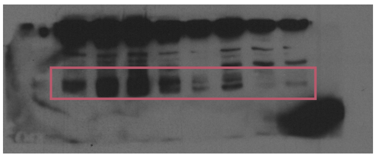

p37 →
p20 →

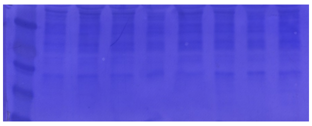

Supplement: S1 Raw images — (PDF) [file pone.0288491.s003.pdf]
